# The Imbalance of Circulating Follicular Helper T Cells and Follicular Regulatory T Cells Is Associated With Disease Activity in Patients With Ulcerative Colitis

**DOI:** 10.3389/fimmu.2020.00104

**Published:** 2020-02-14

**Authors:** Yan Long, Changsheng Xia, Lijuan Xu, Caoyi Liu, Chunhong Fan, Huizhang Bao, Xiaotao Zhao, Chen Liu

**Affiliations:** ^1^Department of Clinical Laboratory, Peking University People's Hospital, Beijing, China; ^2^Department of Immunology, School of Basic Medical Sciences, Peking University Health Science Center, Beijing, China

**Keywords:** ulcerative colitis, follicular helper T cells, follicular regulatory T cells, immune imbalance, disease activity

## Abstract

Ulcerative colitis (UC) is a chronic inflammatory bowel disease affecting the colon and rectum, in which the abnormality of B cells is involved in both its pathogenesis and progression. Follicular helper T cells (TFH) play an important role in assisting the immune function of human B cells in germinal centers, and follicular regulatory T cells (TFR) have the function of inhibiting TFH and germinal center B cell responses. The significance of circulating TFH and TFR in ulcerative colitis (UC) remains unclear. We analyzed peripheral blood of active and stable remission UC patients and found that circulating TFR was significantly decreased while TFH was increased in active UC patients. As to TFH subsets, TFH2 was elevated while TFH17 was decreased in active UC, with IL-4/IL-17A secretion enhanced. Helios^+^ and CD45RA^−^FoxP3^high^ TFR cells were decreased while CD226^+^ and CD45RA^+^FoxP3^int^ TFR cells were increased in active UC patients. The levels of new memory B cells, plasmablasts and serum IgG were significantly increased in active UC patients, and were positively correlated with TFH and TFH2, and negatively correlated with TFR. Serum CRP and Mayo Clinic scores were positively correlated with TFH and TFH2 but negatively correlated with TFR. Serum IL-12 and IL-21 were up-regulated while IL-10 was down-regulated in active UC. To conclude, an imbalance of circulating TFH and TFR cells is associated with disease activity in UC patients. Our results suggest a new mechanism for TFH and TFR imbalance in the pathogenesis of UC, providing a new perspective for theoretical research and therapeutic strategies for UC.

## Highlights

- TFH cells were significantly elevated, and TFH2 cells were predominantly elevated in patients with active UC.
- Peripheral TFR cells were significantly decreased and the proportion of functional subpopulations was also decreased in patients with active UC.
- Changes in TFH and TFR were significantly associated with elevations of new memory B cells and plasmablasts, and elevated serum IgG in UC patients.
- TFH and TFR levels were significantly associated with disease activity of UC patient.
- Serum IL-21, IL-12, and IL-10 cytokines in active UC patients were significantly changed and associated with TFH and TFR levels.

## Introduction

Ulcerative colitis (UC) is a chronic inflammatory bowel disease affecting the colon and rectum and the recurrence of UC appears as alternative between active and remission periods, which has the risk of developing into colitis-related cancer (1, 2). During the active period of UC, the mucosal pathology is expressed as massive neutrophils, lymphocytes and plasma cell infiltration, and lymphoid follicle formation in gut-associated lymphoid tissues (GALT) (3).

An abnormal number and function of B cells caused by the interaction between T and B cells are involved in the pathogenesis of UC (4–8). There are a large number of activated B cells in the lamina propria of UC patients, and a large amount of IgG is expressed in the lesion tissues (6). B cells secreting autoantibodies against colonic epithelial cells could be isolated from the lesions and peripheral blood of UC patients (7). Circulating CD19^+^CD20^−^CD38^+^CD138^+^ plasma cells were also significantly elevated in UC patients, and positively correlated with Mayo clinic scores (8). In summary, abnormal immune activation mediated by B cells plays an important role in the pathogenesis and disease activity of UC.

Accumulated studies have shown that follicular helper T cells (TFH) play an important role in assisting the immune function of human B cells. TFH is differentiated and developed from the initial CD4^+^ T cells in the germinal center (GC), and secretes IL-21 to promote self-proliferation and differentiation (9). TFH cells exist in the germinal center and peripheral blood, and circulating TFH can be divided into TFH1, TFH2, and TFH17 subsets, according to the expression of CXCR3 and CCR6 (10). The main function of TFH is to initiate and regulate germinal center reaction and germinal center B cell maturation and differentiation (11). GC abnormal reaction occurs when the number or function of TFH cells is changed, resulting in the production of a large number of plasma cells and memory B cells, and more high-affinity antibodies and inflammatory factors (11–13). TFH is significantly increased in peripheral blood of systemic lupus erythematosus (SLE) patients and is positively correlated with autoantibody titer and disease activity (14). In rheumatoid arthritis, TFH is significantly elevated and related to disease activity (15). In a DSS UC mouse model, TFH was significantly increased, and positively correlated with the amount of colonic lesions (16). Wang et al. found that the number of CXCR5^+^FoxP3^−^ TFH cells in active UC patients were increased significantly and were negatively correlated with serum CRP and Mayo clinic scores (17). Xue et al. (18) reported that TFH cells and the level of IL-21 were significantly higher in UC patients than in the healthy controls. However, studies of TFH subgroups in UC have not been reported and the significance of TFH in different clinical stages of UC remains unclear.

Follicular regulatory T cells (TFR) are a specific subgroup of Treg expressing CXCR5 and FoxP3, which have been widely studied in recent years. TFR could inhibit TFH and germinal center responses to inhibit humoral immunity. The inhibition of TFR on B cells occurs in B cell differentiation, class switching and plasma cell formation (19–22). The abnormality of number or function of TFR may directly act on B cells or indirectly affect B cells through TFH, causing overactive B cell immune function and contributes to various immune-related clinical diseases (19–22). In SLE patients, the percentage of circulating CD4^+^CXCR5^+^FoxP3^+^TFR and TFR/TFH ratio were significantly increased and positively correlated with disease activity (23). Meanwhile, increased TFR cells were associated with lower autoantibodies in RA patients (24). In terms of UC, there has not been any research on the role or mechanism of TFR in different clinical disease statuses of UC.

In this study, we recruited both UC patients in active phase and stable remission and analyzed circulating TFH and TFR cell levels and their subgroup changes. We aim to clarify the role of TFH and TFR in different phases of UC, which could give new ideas about the prevention and pathogenesis of UC.

## Materials and Methods

### Patients

Eighty-eight UC patients who were consecutively admitted to Peking University People's Hospital from Nov 2018 to April 2019 were enrolled in this study, including both hospitalized patients and outpatients. All patients diagnosed with UC fulfilled the criteria including clinical manifestation, radiographic, endoscopic and histological findings, accordingly (25). The disease activity was evaluated by a Mayo Clinic score. Patients with a Mayo Clinic score >2 were determined to be at active stage, while patients in stable remission were with a Mayo Clinic score ≤ 2 (26). Peripheral blood samples were collected for routine complete blood cell count (CBC) tests and the remaining blood samples were used. Patients who had received any glucorticoid and/or immunosuppressive drugs in the past 3 months, were diagnosed with other autoimmune diseases or tumors or indeterminate Colitis and Crohn's disease, or with intestinal infectious diseases or allergic diseases, were excluded. Clinical data including Mayo score, age, gender, serum CRP, and IgG levels etc. were collected from hospital records retrospectively. Forty-four cases of healthy adults were enrolled as healthy controls (HC) from Physical Examination Center.

### Patients' Follow-Up

In the 44 active UC patients, 27 patients had achieved clinical remission after being followed up for 8–12 weeks, and 17 patients failed to be followed up. Among the 27 patients, 11 were treated with 5-aminosalycilates (5-ASA) and the others were treated with corticosteroids or thiopurines. We followed up 11 patients achieving clinical remission through treatment with 5-ASA, and patients using corticosteroids or thiopurines were not analyzed because there may be impact on lymphocytes. We collected blood samples of the 11 followed-up patients, and the levels of TFH/TFR subsets were analyzed.

### Flow Cytometry

Peripheral blood mononuclear cells (PBMCs) were isolated by Ficoll separation (Ficoll-Paque, Pharmacia, Sweden). Cells were washed and incubated for 30 min with fluorescent antibodies for surface markers. All antibodies used were purchased from BioLegend (San Diego, CA, USA). After being washed twice in PBS, cells were intracellularly stained using FoxP3 Staining Buffer Kit (eBioscience, San Diego, CA, USA), and then incubated with anti-FoxP3 allophycocyani for 30 min. For Helios or intracellular cytokine analysis, anti-Helios FITC, or anti-IL-4 FITC or anti-IL-17A FITC were added. After being washed, samples were analyzed on FACSCanto using Diva software (BD Biosciences, San Jose, CA, USA).

### *In vitro* Culture

PBMCs were cultured with PMA (50 ng/ml) and ionomycin (1 mg/ml) in RPMI 1640 medium containing 10% FBS for 5 h in 96 well flat-bottom plates at 37°C using 5% CO_2_ incubator, with brefeldin A (3 mg/ml; Sigma-Aldrich) in the complete medium. Cells were then harvested, stained and analyzed by Flow cytometry.

### ELISA

IL-4, IL-17A, IL-21, IL-12p70, and IL-10 concentrations in serum were measured using ELISA Kits from Biolegend (San Diego, CA, USA), according to the manufacturer's instruction. Their concentrations were calculated using standard curves and each sample was measured in duplicate.

### Statistical Analysis

Data were presented as mean ± SD. The differences between each pairs among three groups were analyzed by One-way ANOVA test with Bonferroni's adjustment. The differences of TFH/TFR subsets in follow-up patients between pre-treatment and post-treatment were analyzed using paired *t*-test. The correlation analyses between TFH/TFR subsets and clinical indicators, cytokines were conducted using the Spearman test. All statistical tests were 2-tailed and the *p*-values < 0.05 were considered to be statistically significant. All these analyses were conducted using GraphPad Prism (GraphPad Software, San Diego, CA, USA).

## Results

### Circulating TFH Cells Were Increased While TFR Cells Were Decreased in Active UC Patients

**Figure 1:**
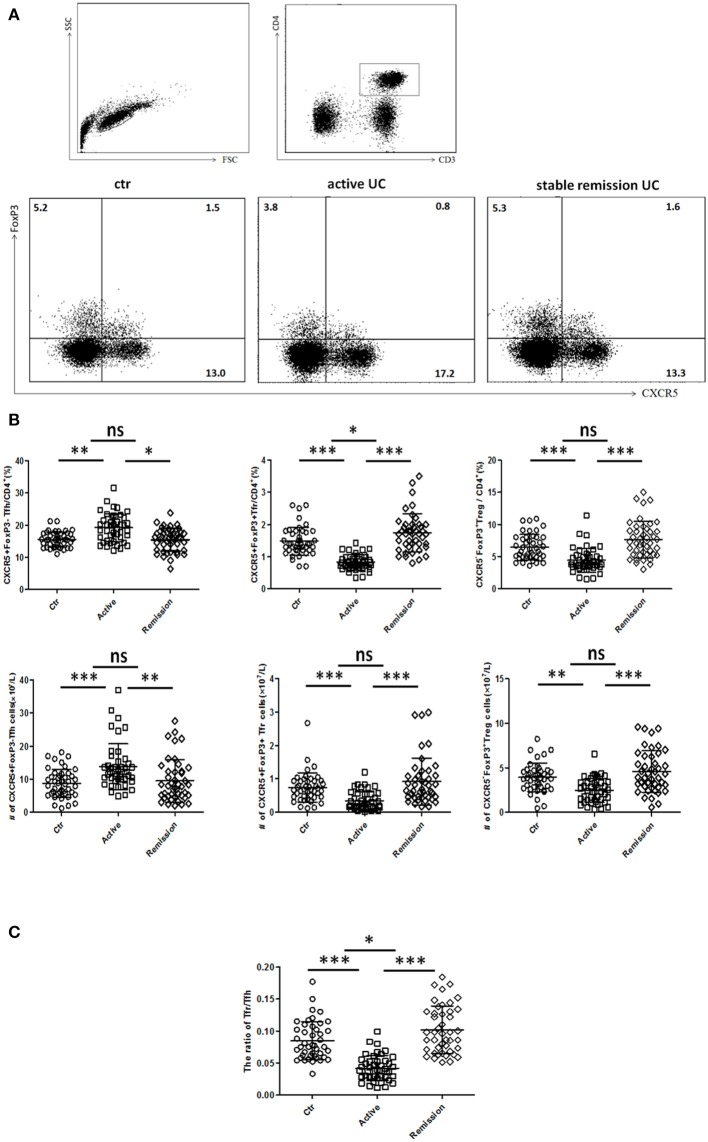
Flow cytometry analysis of circulating TFR and TFH in UC patients and healthy controls. Peripheral blood from UC patients in active stage (*n* = 44), stable remission (*n* = 44) and HC (*n* = 44) was collected, and then TFH, TFR cells were measured by FACS through staining of CD3, CD4, CXCR5, and FoxP3. **(A)** Representative dot plots for flow cytometry used in this study. TFR, TFH, and Treg were defined as: CD4^+^CXCR5^+^FoxP3^+^cells (TFR), CD4^+^CXCR5^+^FoxP3^−^ cells (TFH), and CD4^+^CXCR5^−^FoxP3^+^ cells (Treg). Numbers show the percentage of TFR, TFH, and Treg in CD4^+^ lymphocytes. **(B)** Comparison of average percentage (up) and absolute numbers (bottom) of TFR, TFH, and Treg among three groups. **(C)** The comparison of ratios of CD4^+^CXCR5^+^FoxP3^+^ TFR cells to CD4^+^CXCR5^+^FoxP3^−^ TFH cells in three groups. Symbols represent individual subjects and bars show the mean ± SD. *p < 0.05; **p < 0.01; ***p < 0.001; ns, not significant.

We included 88 UC patients and 44 healthy controls. Clinical characters of all the individuals were shown in Table S1 in Supplementary Material. CD3^+^CD4^+^CXCR5^+^FoxP3^−^ TFH, CD3^+^CD4^+^CXCR5^+^FoxP3^+^ TFR, and CD3^+^CD4^+^CXCR5^−^FoxP3^+^ Treg cells were analyzed (Figure 1). We found both percentage and absolute number (per liter) of circulating TFH cells were significantly increased in active UC patients. However, the percentage and absolute number of circulating TFR were significantly lower in active UC patients, compared with HC and stable remission UC patients. In addition, the levels of CD3^+^CD4^+^CXCR5^−^FoxP3^+^ Treg cells were decreased in active UC (Figure 1B). We calculated the ratio of TFR/TFH, and found it was significantly lower in active UC patients, compared with HC and stable remission UC patients (Figure 1C).

### CXCR3^−^CCR6^−^TFH2 Subsets Were Increased and CXCR3^−^CCR6^+^TFH17 Subsets Were Decreased in Active UC Patients

**Figure 2:**
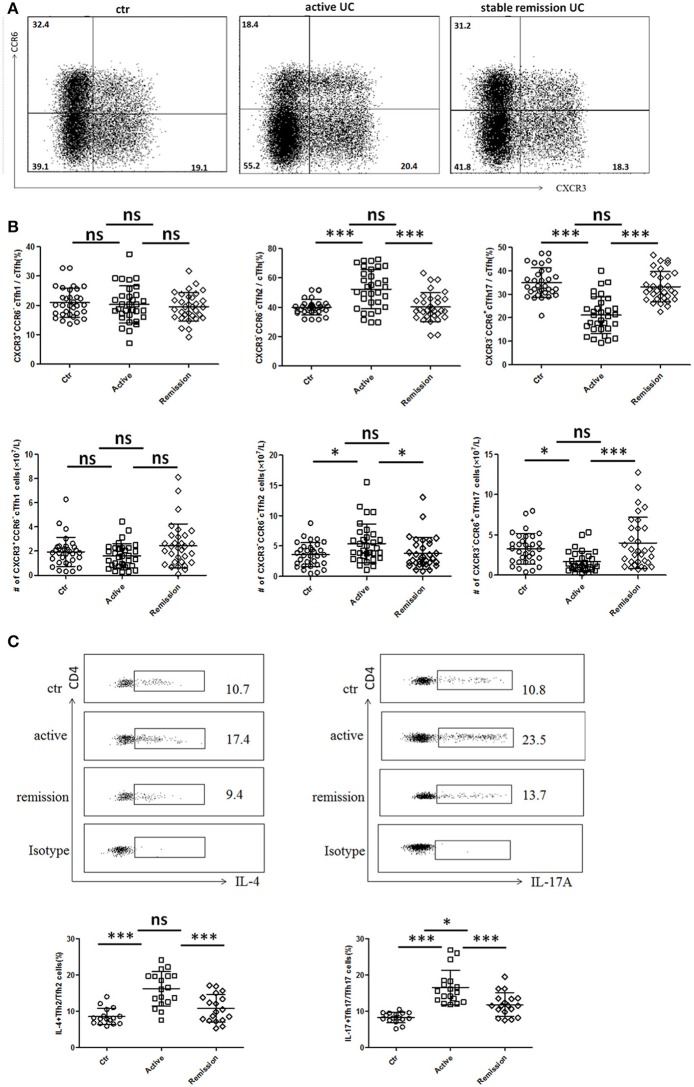
Flow cytometry analysis of TFH subset levels and cytokine secretion in UC patients. **(A,B)** Peripheral blood from active UC patients (*n* = 32), stable remission UC patients (*n* = 32), and HC (*n* = 32) was collected and TFH subsets were analyzed. TFH1, TFH2, and TFH17 were defined as follows: TFH1 (CD3^+^CD4^+^CXCR5^+^FoxP3^−^CXCR3^+^CCR6^−^), TFH2 (CD3^+^CD4^+^CXCR5^+^FoxP3^−^CXCR3^−^CCR6^−^), and TFH17 (CD3^+^CD4^+^CXCR5^+^FoxP3^−^CXCR3^−^ CCR6^+^). **(A)** Representative cytometry strategy for detection of CXCR3 and CCR6 expressions in CD3^+^CD4^+^CXCR5^+^ FoxP3^−^ T cells. Numbers indicate the percentage of cells in each quadrant. **(B)** The comparison of average percentage (up) and absolute number per liter (bottom) of TFH1, TFH2, TFH17 subsets among active UC patients, patients in stable remission UC and HC. **(C)** Peripheral blood from active UC patients (*n* = 18), stable remission UC patients (*n* = 18) and HC (*n* = 16) were incubated with PMA, ionomycin and BFA for 5 h and cytokines within TFH subsets were analyzed by intracellular staining. Representative dot plots of IL-4 secretion levels in TFH2 subset and IL-17A secretion levels in TFH17 subset. Numbers show the percentages of IL-4^+^ cells among TFH2 and IL-17A^+^ cells among TFH17 lymphocytes (up). IL-4^+^ cell percentages in TFH2 and IL-17A^+^ cells among TFH17 were compared (bottom), respectively. All Symbols represent individuals and bars show the mean ± SD. *p < 0.05; ***p < 0.001; ns, not significant.

Circulating TFH can be divided into TFH1 (CXCR3^+^CCR6^−^), TFH2 (CXCR3^−^CCR6^−^), and TFH17 (CXCR3^−^CCR6^+^) subsets, and each TFH subsets also have significant differences in cytokines secretion (10, 27). We analyzed 32 cases in active stage and 32 cases in stable remission subsequently enrolled in this study, respectively. As shown in Figures 2A,B, higher percentages of circulating CXCR3^−^CCR6^−^TFH2 cells and lower levels of CXCR3^−^CCR6^+^TFH17 cells were found in active UC patients, compared with UC patients in stable remission and HC. We calculated absolute number (per liter) and got similar results. TFH1 cells were not significantly different among three groups (Figure 2B).

We studied cytokine secretion changes in TFH2 and TFH17 by *in vitro* culture. As shown in Figure 2C, IL-4 secretion of TFH2 was strengthened in active UC patients. IL-17A secretion of TFH17 in active UC patients was also stronger, though TFH17 level was decreased. Meanwhile, the IL-17A secretion was also enhanced in stable remission UC patients, compared with HC.

We also found IL-4 was significantly elevated and IL-17A was increased in active UC patients, while UC patients in stable remission was not significantly changed compared with HC (Figure S1A in Supplementary Material). Considering that serum IL-4 and IL-17A can also be secreted by Th2 and Th17, we examined circulating IL-4^+^Th2 and IL-17A^+^Th17 and found both frequencies and absolute numbers of IL-4^+^Th2 and IL-17A^+^Th17 were increased in active UC patients (Figure S1B in Supplementary Material). We speculated that in active UC, TFH2, and Th2 are simultaneously increased, and the IL-4 secretion of TFH2 was also enhanced, leading to elevated serum IL-4. TFH17 was decreased while Th17 was increased, but the IL-17A secretion of TFH17 cells is enhanced, and the combined effects of the changes in TFH17 and Th17 resulted in an increase in serum IL-17A. Meanwhile, we also tested the IFN γ secretion ability of TFH1 cells in some UC patients by *in vitro* stimulation, but we found that it was consistent with the unchanged TFH1 level in UC patients. The change of IFN γ secreted by TFH1 cells in UC patients was not significant (data not shown).

### Helios Expression Was Significantly Decreased While CD226 Expression Was Obviously Increased in TFR Cells in Active UC Patients

**Figure 3:**
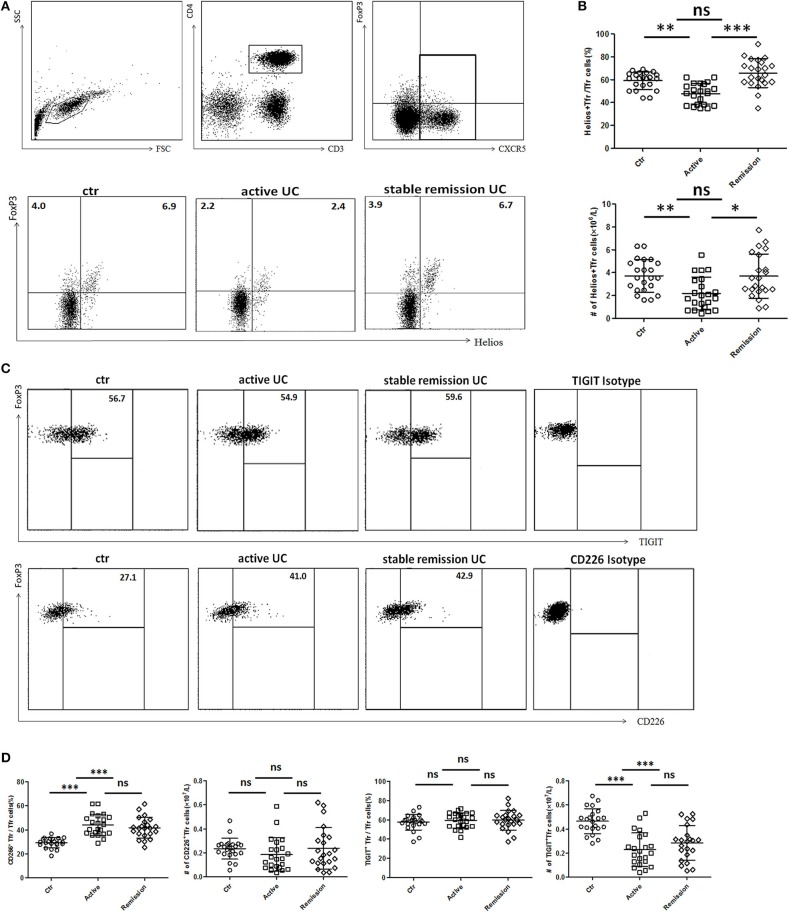
Comparison of functional subsets of TFR from UC patients and healthy controls (HC) according to Helios, TIGIT, and CD226. Peripheral blood from UC patients subsequently enrolled in this research, including active UC patients (*n* = 22) and stable remission UC patients (*n* = 22) and HC (*n* = 22) were collected and functional subsets of TFR cells were analyzed through staining for CD3, CD4, CXCR5, FoxP3, Helios, TIGIT, and CD226. **(A)** Representative dot plots for Helios and FoxP3 analysis. Numbers represent the percentages among CD3^+^CD4^+^CXCR5^+^ subsets. **(B)** Percentage of Helios^+^ TFR cells among CD3^+^CD4^+^CXCR5^+^FoxP3^+^ TFR cells (up) and absolute number of Helios^+^ TFR cells per liter in active or stable remission UC and HC (bottom). **(C)** Representative dot plots for analyzing expression of TIGIT (up) or CD226 (bottom) among TFR. Numbers represent percentages of TIGIT or CD226 positive cells in CD3^+^CD4^+^CXCR5^+^FoxP3^+^ TFR cells. **(D)** Percentages and absolute numbers of CD226^+^ TFR or TIGIT^+^ TFR subsets among TFR cells were compared among active UC patients, stable remission UC patients and HC. All symbols represent individual subjects and bars show the mean ± SD. *p < 0.05; **p < 0.01; ***p < 0.001; ns, not significant.

We further analyzed functional subpopulations of TFR to reflect TFR functional changes in 22 cases of active UC patients and 22 cases in stable remission subsequently enrolled in the whole subjects. Helios^+^ Treg cells were reported to have a stronger immunosuppressive function (28, 29). T cell Ig and ITIM domain (TIGIT) is associated with suppressive capacity of Treg cell and the CD226^+^ Treg is associated with reduced Treg suppressive capacity (30, 31). We found significantly lower percentages and absolute number (per liter) of Helios^+^ TFR cells in active UC patients (Figures 3A,B). Higher percentages of CD226^+^ TFR cells were observed in both active and stable remission UC patients than HC (Figures 3C,D). The absolute numbers of TIGIT^+^ TFR cells were greatly decreased in UC patients, compared with HC (Figure 3D). We also found that percentages of CD226^−^ TIGIT^+^ TFR cells were decreased while CD226^+^TIGIT^−^ TFR cells were increased in active UC patients, compared with HC (Figure S2 in Supplementary Material). We analyzed TFR subpopulations according to the expression of CD45RA and FoxP3 (32). We found significantly decreased circulating CXCR5^+^CD45RA^−^FoxP3^high^ activated TFR cell percentages and absolute numbers and increased CD45RA^+^FoxP3^int^ resting TFR cell percentages in active UC patients (Figure S3 in Supplementary Material). These results suggest that TFR cells in active UC patients are with a weakened inhibition function.

### Serum IgG, Circulating New Memory B Cells, and Plasmablasts Were Increased and Correlated With TFH and TFR Levels

**Figure 4:**
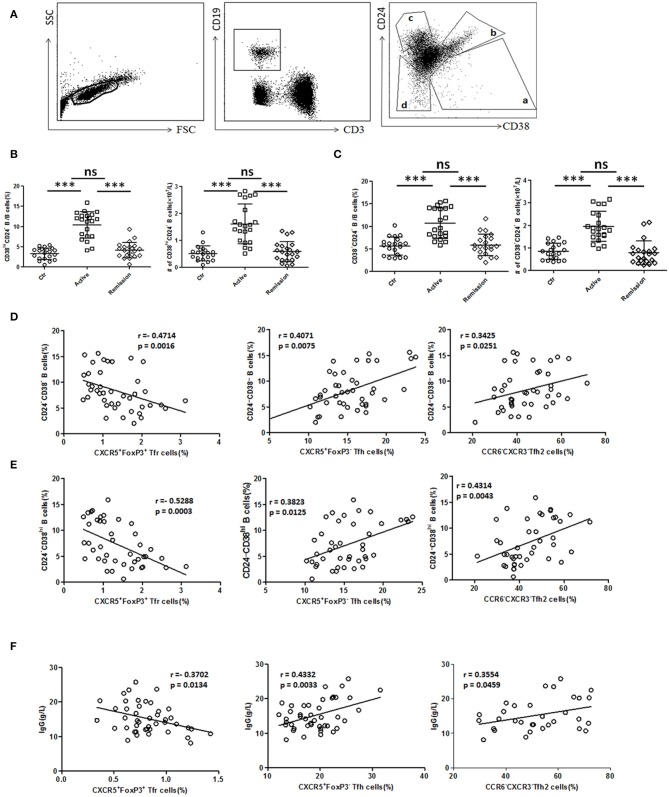
B cell subset and serum IgG analysis and their relationship with TFR, TFH, and TFH2. **(A)** Peripheral blood from active UC patients (*n* = 21), stable remission UC patients (*n* = 21), and HC (*n* = 20) were collected. CD24 and CD38 expression classifies circulating B cells into primarily memory B cells (CD19^+^CD38^−^CD24^high^), plasmablasts (CD19^+^CD38^high^CD24^−^), transitional (CD19^+^CD38^high^CD24^high^), and new memory (CD19^+^CD24^−^CD38^−^) B cell subsets, marked sequentially a–d. Representative dot plots for staining for CD19, CD3, CD24, and CD38 was shown. **(B,C)** Percentages and absolute numbers (per liter) of new memory (d subset) and plasmablasts (a subset) were compared among three groups. Symbols represent individual subjects and bars show the mean ± SD. ***p < 0.001; ns, not significant. **(D,E)** Correlation analysis between TFR, TFH, TFH2, and new memory B cells or plasmablasts was carried out in all UC patients (*n* = 42, including 21 active and 21 in stable remission). **(F)** Serum IgG levels were analyzed and correlation analysis were conducted between IgG and TFR, TFH levels in 44 active UC patients, and between IgG and TFH2 levels in 32 active UC patients. The *r*-values were the Spearman's correlation coefficients, and *p* < 0.05 was linearly regressed to show relevant trends.

We next analyzed functional B cell subsets and studied their relationships with TFH and TFR. The diversity of CD24 and CD38 expressions classifies circulating B cells into primarily memory B cells (CD19^+^CD38^−^CD24^high^), plasmablasts (CD19^+^CD38^high^CD24^−^), new memory B cells (CD19^+^CD24^−^CD38^−^) and transitional B cells (CD19^+^CD38^high^CD24^high^) (33). We found both percentage and absolute number of new memory B cells and plasmablasts were significantly increased in active UC patients (Figures 4A,B,C). In UC patients, both new memory B cells and plasmablasts were positively correlated with TFH (*r* = 0.4071, *p* = 0.0075; *r* = 0.3823, *p* = 0.0125, respectively), TFH2 (*r* = 0.3425, *p* = 0.0251; *r* = 0.4314, *p* = 0.0043, respectively), and negatively correlated with TFR (*r* = −0.4714, *p* = 0.0016; *r* = −0.5288, *p* = 0.0003, respectively) (Figures 4D,E).

Furthermore, we found that serum IgG levels in active UC patients were also significantly higher than those in stable remission UC patients and HC (Figure S4A in Supplementary Material). We found IgG concentrations were positively correlated with TFH (*r* = 0.4332, *p* = 0.0033), TFH2 (*r* = 0.3554, *p* = 0.0459), and negatively correlated with TFR (*r* = −0.3702, *p* = 0.0134) in active UC patients (Figure 4F). In addition, we also analyzed all of the UC patients as a whole, and performed the correlation analysis between their serum IgG levels and TFH (*r* = 0.4201, *p* < 0.0001), TFH2 (*r* = 0.4080, *p* = 0.0008), and TFR (*r* = −0.5307, *p* < 0.0001), and obtained consistent results (Figure S4B in Supplementary Material). These results suggest that TFH and TFR changes are related to increased memory B cells, plasmablasts and up-regulated serum IgG in active UC patients.

### TFH and TFR Were Correlated With Disease Activity and Serum CRP of UC Patients

**Figure 5:**
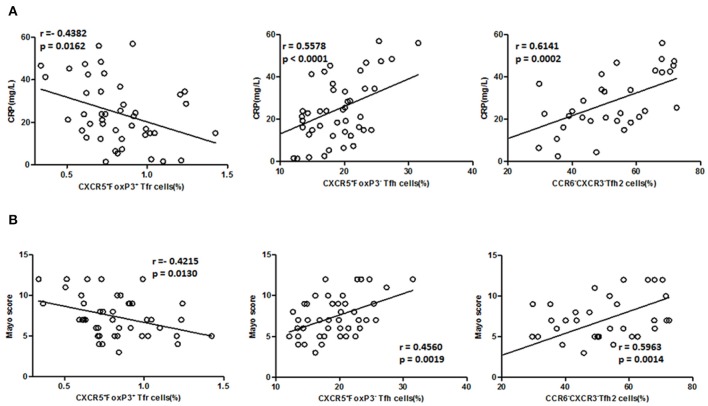
Correlation between TFR, TFH, TFH2 subsets, and clinical parameters in active UC patients. **(A)** Levels of CRP in active UC patients were measured, and correlation analysis with percentages of CD4^+^CXCR5^+^FoxP3^+^ TFR cells (*n* = 44), CD4^+^CXCR5^+^FoxP3^−^ TFH cells (*n* = 44), CXCR3^−^CCR6^−^ TFH2 subsets (*n* = 32) were performed. **(B)** The disease activity of UC was evaluated by Mayo Clinic Score, and correlations with TFR and TFH cells in active UC patients (*n* = 44), and with percentages of TFH2 cells in active UC patients (*n* = 32) were performed. The r-values were the Spearman's correlation coefficients, and *p* < 0.05 was linearly regressed to show relevant trends.

We further analyzed the association of TFH and TFR with the clinical indicators of UC patients (Figures 5A,B). In active UC, serum CRP levels and Mayo scores were significantly negatively correlated with TFR levels (*r* = −0.4382, *p* = 0.0162; *r* = −0.4215, *p* = 0.0130, respectively) and significantly positively correlated with TFH (*r* = 0.5578, *p* < 0.0001; *r* = 0.4560, *p* = 0.0019, respectively) and TFH2 levels (*r* = 0.6141, *p* = 0.0002; *r* = 0.5963, *p* = 0.0014, respectively). We also did correlation analysis between serum CRP levels and TFH (*r* = 0.5782, *p* < 0.0001), TFH2 (*r* = 0.4202, *p* = 0.0005) and TFR (*r* = −0.5776, *p* < 0.0001) in UC patients in both active stage and in stable remission, and obtained consistent results (Figure S5 in Supplementary Material). These results indicate that changes in TFH and TFR were associated with clinical conditions of UC patients.

### TFH and TFR Subsets Were Recovered in Patients Achieving Stable Remission From Active Stage

**Figure 6:**
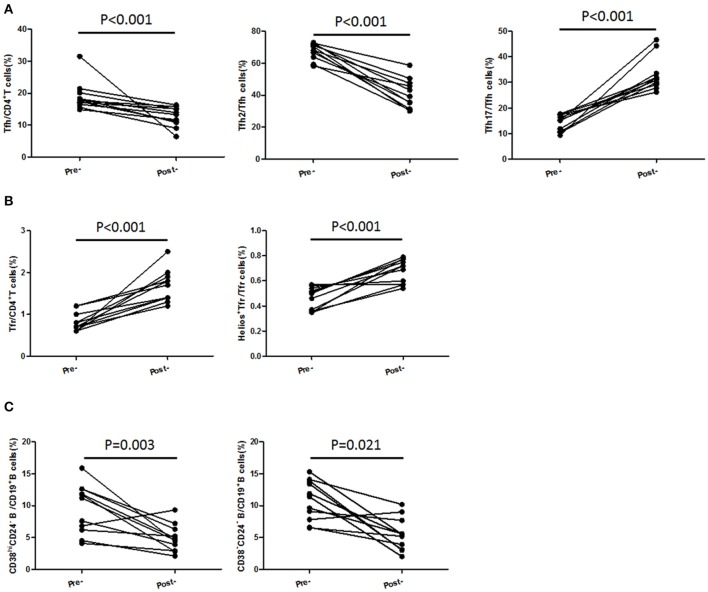
TFH and TFR subsets were recovered in patients achieving stable remission from active stage. Peripheral blood were collected from 11 active UC patients who achieved stable remission after being treated with 5-ASA. **(A,B)** CD4^+^CXCR5^+^FoxP3^+^ TFR cells, CD4^+^CXCR5^+^FoxP3^−^ TFH cells, CD3^+^CD4^+^CXCR5^+^FoxP3^−^CXCR3^−^CCR6^−^TFH2 cells, CD3^+^CD4^+^CXCR5^+^FoxP3^−^CXCR3^−^ CCR6^+^ TFH17 cells, and Helios+ TFR cells were analyzed by flow cytometry and compared with their levels before treatment. **(C)** B cell subsets were analyzed by staining with CD19 CD24 and CD38 antibodies. Plasmablasts (CD19^+^CD38^high^CD24^−^) and new memory B cells (CD19^+^CD24^−^CD38^−^) were calculated and compared with their levels before treatment.

In 11 followed-up active UC patients treated with 5-ASA, we observed a significant decrease of TFH and recovery of TFR when achieving clinical remission after treatment with 5-ASA (Figures 6A,B). As to TFH and TFR subsets, we found a significant decrease of CXCR3^−^CCR6^−^TFH2 subsets and elevation of Helios^+^ TFR percentages and CXCR3^−^CCR6^+^TFH17 subsets in patients achieving stable remission (Figures 6A,B). In addition, we also found the levels of CD38^high^CD24^−^ plasmablasts and CD38^−^CD24^−^ new memory B cells were significantly decreased after achieving stable remission (Figure 6C).

### Changes of TFH-Associated Cytokines in UC Patients

**Figure 7:**
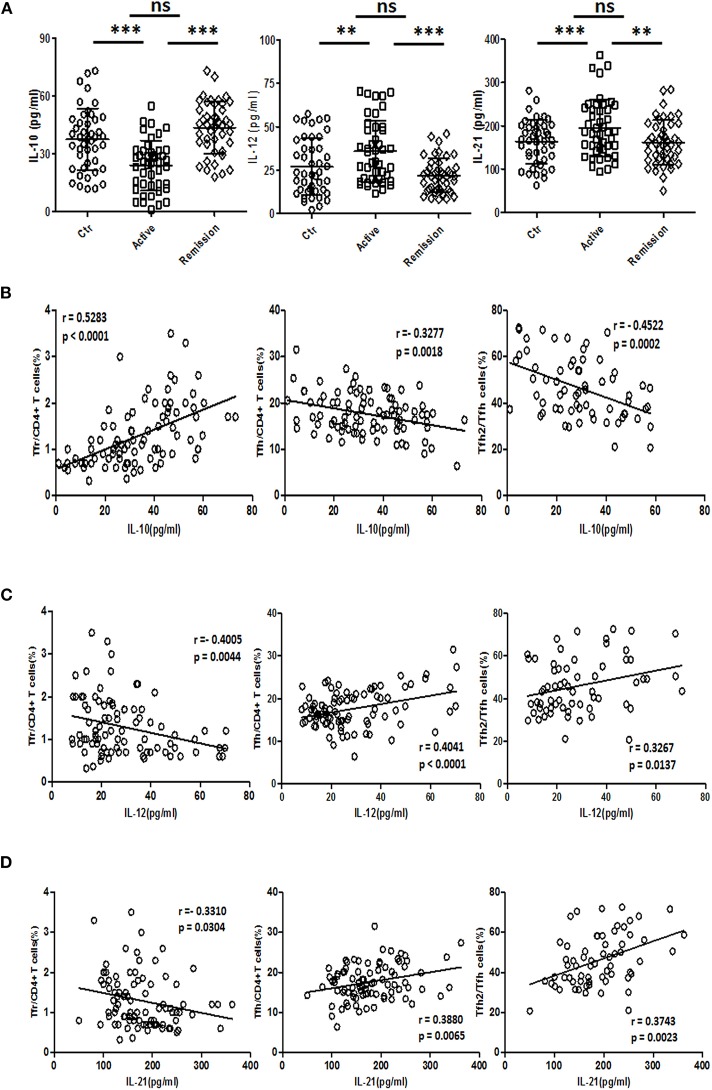
Serum IL-10, IL-12, and IL-21 analysis and their relationship with TFR and TFH. **(A)** IL-10, IL-12, and IL-21 levels in serum of 44 healthy controls, 44 UC patients in active stage and 44 UC patients in stable remission were analyzed by ELISA. Their levels among three groups were compared. Symbols represent individual subjects and bars show the mean ± SD. **p < 0.01; ***p < 0.001; ns, not significant. **(B–D)** Correlation analysis between serum IL-10, IL-12, IL-21 concentrations, and percentages of CXCR5^+^FoxP3^+^ TFR cells, CXCR5^+^FoxP3^−^ TFH cells was performed among 88 UC patients, including 44 active and 44 stable remission UC patients. Correlation analysis between cytokine levels and CXCR3^−^CCR6^−^ TFH2 subsets was conducted in 64 UC patients (32 active and 32 stable remission UC patients). The r-values were the Spearman's correlation coefficients, and *p* < 0.05 was linearly regressed to show relevant trends.

IL-21 and IL-12 were reported to promote the differentiation of TFH cells (34, 35), while IL-10 is a negative regulator of TFH (36). We analyzed the concentrations of IL-12 (IL-12p70), IL-21, and IL-10 in serum. IL-10 levels in serum were decreased in active UC patients, compared with HC and stable remission UC patients. And both serum IL-12 and IL-21 were significantly higher in active UC (Figure 7A).

Correlation studies were conducted in all UC patients. The results showed that IL-10 concentration was negatively correlated with TFH (*r* = −0.3277, *p* = 0.0018) and TFH2 (*r* = −0.4522, *p* = 0.0002), and positively correlated with TFR (*r* = 0.5283, *p* < 0.0001) (Figure 7B), while IL-12 was positively correlated with TFH (*r* = 0.4041, *p* < 0.0001) and TFH2 (*r* = 0.3267, *p* = 0.0137), but negatively correlated with TFR (*r* = −0.4005, *p* = 0.0044) (Figure 7C). IL-21 was positively correlated with TFH (*r* = 0.3880, *p* = 0.0065) and TFH2 (*r* = 0.3743, *p* = 0.0023) and negatively correlated with TFR (*r* = −0.3310, *p* = 0.0304) (Figure 7D). The correlations between these three cytokines and the levels of TFH17 were not significant (data not shown). We also did correlation analysis only in active UC patients, and found that IL-12 and IL-21 were significantly positively correlated with TFH (*r* = 0.3458, *p* = 0.0215; *r* = 0.4342, *p* = 0.0032, respectively), but not correlated with TFR. However, the correlation between IL-10 and TFH was not significant, but it was significantly positively correlated with TFR (*r* = 0.3328, *p* = 0.0273) (Figure S6 in Supplementary Material).

## Discussion

In this study, we systematically described the changes in circulating TFH and TFR cells in UC. We found that peripheral TFR cells were significantly decreased and functional TFR subsets were also decreased in active UC, while TFH cells were significantly elevated and the composition of TFH were altered with a predominant increase of TFH2 and a significant decrease of TFH17. Furthermore, the changes in TFH and TFR were significantly associated with increased memory B cells and plasmablasts, and elevated serum IgG. TFH and TFR were significantly associated with the disease severity.

So far, there have been relatively few studies on the role of TFH and TFR in UC. Yu et al. (16) reported that IL-21 could regulate the proliferation and response of TFH cells in the colitis microenvironment. Sebastian Fuchs et al. found that the frequency of CXCR5^+^PD1^+^TFH cells in peripheral blood was significantly increased in 5-ASA treated UC patients (37). Decreased circulating FoxP3^+^CXCR5^+^ TFR cells and increased FoxP3^−^CXCR5^+^TFH cells in UC patients were once reported (17), but their study lacked detailed clinical staging studies. In addition, changes in the detailed functional subsets of TFH and TFR, and the relationship between TFH/TFR and B cell subsets and serum IgG have not been elucidated. The staging of UC can reflect the effects of TFH and TFR cells in UC more deeply. In addition to level changes, the functions of TFH and TFR are more important. Considering the difficulty to sort sufficient number of TFR for *in vitro* culture, we used functional subpopulation analysis to reflect functional changes, since these subpopulations have been previously demonstrated. For the subpopulations that reflect the function of TFR cells, our results indicate that there are some subpopulations that are different between active and stable remission UC patients, but some are not different. This reflects that the state of TFR cells in the blood of UC patients in stable remission is in a transition state between active UC and healthy people, which also reflects the role of TFR cells in inhibiting the progression of UC disease to a certain extent. In addition, the analysis of memory B cell and plasmablast levels combined with changes in TFH can provide us a better understanding of the role of TFH and TFR in UC.

**Figure 8:**
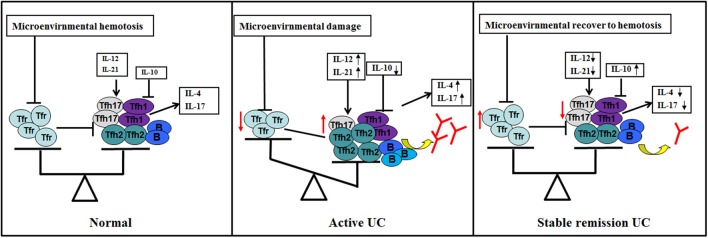
Hypothetical schematic of the study. TFR and TFH and subgroups (TFH1/TFH2/TFH17) in normal human body (GALT and peripheral blood) are in equilibrium (left). When UC occurs and the disease is active (middle), (1) changes in microenvironment caused by certain factors, such as antigen or intestinal flora lead to a decrease in the number and/or function of TFR by some pathways; (2) changes in cytokines, such as IL12, IL-21, and IL-10 in the microenvironment also act on TFH to promotes elevated levels and subpopulation changes of TFH; (3) TFR and changes in cytokines act on TFH and act indirectly and/or directly on B cells; (4) B cells (memory B or plasma cells) have enhanced immune function, resulting in increased serum antibody levels and active clinical stage; (5) when the microenvironment of the body recovers, level and/or function of TFR and TFH are restored, TFR restores normal inhibition of excessive serum antibody production of B cell, helping UC patients progress from active to stable remission (right).

Based on current results, we propose new ideas for the role of TFH and TFR in UC (Figure 8). Under normal conditions, TFH and TFR are in equilibrium and B cells secrete antibodies moderately; when UC occurs, the intestinal and peripheral blood microenvironment changes, and TFR levels are reduced. Decreased TFR and changed cytokines result in elevated TFH and an imbalance of TFH and TFR. Increased B cells or plasma cells produce higher immunoglobulin, leading to an acute exacerbation of the disease. When the patient is treated, the microenvironment recovers and the balance of TFR and TFH is restored. Immunoglobulin is correspondingly decreased to normal levels, and the patient enters a stable remission state.

However, it may take a lot of work to fully prove such an idea. Because of the ethical limitations in human trials, we were unable to perform germinal-center-related tests. Therefore, animal models may be used for further research to test TFH and TFR changes in the intestine germinal centers. Another question is whether changes in TFH drive or reflect disease status, which is also a key issue. But the current research could not answer this question well, because our current research is, on the whole, an observational study in patient groups, and does not involve intervention, which is not ethically feasible at the present research stage. Therefore, we will conduct mice experiments, in which the effects of TFH and TFR cells can be discussed in detail.

We propose that the increase of IL-21 and IL-12 and the decrease of IL-10 may have an effect on the increase of TFH, but it is not yet clear whether these are the direct causes of the increase of TFH, since the reduction of TFR cells can also result in increase of TFH. In addition, the increase in TFH produced in germinal center can also lead to more peripheral TFH cells. Therefore, animal experiments are needed. The reason for the decrease of TFR cells has not been explored. Studies have shown that PD-1/PD-L1 signaling can reduce the number of TFR in peripheral blood (38). After CTLA-4 knockout, the number of TFR increased significantly, suggesting CTLA-4 may plays an important role in TFR development (39). In-depth studies of these signaling pathways could help to reveal the reasons for the decrease in TFR cells during active UC.

This study still has limitations. The number of patients should be greatly increased or multi-center research should be carried out to better support our conclusions. The detailed mechanisms of TFH and TFR changes, as well as the changes of TFH and TFR in intestinal tissues, need to be elucidated in future. In summary, our results suggest a new mechanism for TFH and TFR imbalance in the pathogenesis of UC, providing a new perspective for theoretical research and therapeutic strategies for UC.

## Data Availability Statement

The raw data supporting the conclusions of this article will be made available by the authors, without undue reservation, to any qualified researcher.

## Ethics Statement

The studies involving human participants were reviewed and approved by the Ethics Committee of Peking University People's Hospital. The patients/participants provided their written informed consent to participate in this study.

## Author Contributions

ChL took charge of all the work and participated in its design. YL carried out most of the experiments and drafted the manuscript. CX and CF did cellular experiments. HB and LX did the clinical measurements. CaL was in charge of analysis of data. XZ contributed to the concept as well. All the authors had approved the final draft submitted.

### Conflict of Interest

The authors declare that the research was conducted in the absence of any commercial or financial relationships that could be construed as a potential conflict of interest.

## Abbreviations

BFA: brefeldin A
CBC: complete blood cell count
CRP: C-reactive protein
GALT: gut-associated lymphoid tissues
GC: germinal center
HC: healthy controls
Ig: immunoglobulin
IL: interleukin
PBMC: peripheral blood mononuclear cells
PMA: phorbol myristate acetate
SLE: systemic lupus erythematosus
SLEDAI: SLE Disease Activity Index
TFH: follicular helper T cells
TFR: follicular regulatory T cells
TIGIT: T cell Ig and ITIM domain
Treg: regulatory T cells
UC: ulcerative colitis
WBC: white blood cells
5-ASA: 5-aminosalycilates.
