# Supplementary material for: The Imbalance of Circulating Follicular Helper T Cells and Follicular Regulatory T Cells Is Associated With Disease Activity in Patients With Ulcerative Colitis
Source: Front Immunol. 2020 Feb 14;11:104. doi: 10.3389/fimmu.2020.00104 (PMC7034313; doi:10.3389/fimmu.2020.00104)
Supplement: Supplementary file 1 [file Data_Sheet_1.docx]

**Figure S1. Serum levels of IL-4 and IL-17A and circulating IL-4^+^Th2 and IL-17A^+^Th17 analysis among UC patients**

A. Serum levels of both IL-4 and IL-17A were measured in serum of three groups, including active UC patients (n=44), UC patients in stable remission (n=44) and healthy controls (n=44) by ELISA.

B. Peripheral blood was incubated with PMA, ionomycin and BFA for 5h and cytokines within CD4^+^ T cells were analyzed by intracellular staining. Percentages (up) and absolute numbers per liter (bottom) of IL-4^+^Th2 and IL-17A^+^Th17 subsets were compared among active UC patients (n=18), stable remission UC patients (n=18) and healthy controls (n=16).

**Figure S2. Comparison of subgroups of circulating TFR cells from UC patients and healthy controls (HC) according to TIGIT and CD226**

Peripheral blood from UC patients, including active UC patients (n=22) and stable remission UC patients (n=22) and healthy controls (n=22) were collected and functional subsets of TFR cells were analyzed through staining for CD3, CD4, CXCR5, FoxP3, TIGIT and CD226.

A. Representative dot plots for analyzing expression of both TIGIT and CD226 among TFR. Numbers in each quadrant represent percentages in CD3^+^CD4^+^CXCR5^+^FoxP3^+^ TFR cells.

B. Percentages and absolute numbers (per liter) of CD226^+^TIGIT^-^ TFR or CD226^-^TIGIT^+^ TFR subsets among TFR cells were compared among active UC patients, stable remission UC patients and healthy controls.

All symbols represent individual subjects and bars show the mean±SD. *, *p* < 0.05; **, *p* < 0.01; ***, *p* < 0.001; ns, not significant.

**Figure S3. TFR subsets analysis for UC patients and healthy controls according to CD45RA and FoxP3**

Peripheral blood from 22 cases of active UC patients, 22 cases of stable remission UC patients and 22 healthy controls subsequently recruited were stained with fluorescent antibodies to CD3, CD4, CXCR5, CD45RA, and FoxP3 and subsets of TFR cells were analysis according to expression of CD45RA and FoxP3.

A. Representative dot plots of CD45RA and FoxP3 expression within the CD3^+^CD4^+^CXCR5^+^FoxP3^+^ TFR cells for three groups. Numbers show the percentages of CD45RA^-^FoxP3^hi^TFR cells, CD45RA^-^FoxP3^int^ TFR cells and CD45RA^+^FoxP3^int^ TFR cells.

B. Percentage and absolute number (per liter) of CD45RA^-^FoxP3^hi^ TFR cells, CD45RA^-^FoxP3^int^ TFR cells and CD45RA^+^FoxP3^int^ TFR cells were compared among the three groups of individuals.

Symbols represent individual subjects and bars show the mean±SD. **, *p* < 0.01; ***, *p* < 0.001; ns, not significant.

**Figure S4. Comparison of IgG between UC patients and healthy controls and correlation analyses of IgG with TFH, TFR among all UC patients**

A. Levels of IgG in serum of active UC patients (n = 44), stable remission UC patients(n=44) and healthy controls(n=44) were measured and compared. All symbols represent individual subjects and bars show the mean±SD. ***, *p* < 0.001; ns, not significant.

B. Correlation analysis with percentages of CD4^+^CXCR5^+^FoxP3^+^ TFR cells(n=88), CD4^+^CXCR5^+^FoxP3^-^ TFH cells(n=88), CXCR3^-^CCR6^-^ TFH2 subsets(n=64) were performed in both active and stable remission UC patients. Spearman’s correlation coefficients are shown. Data are presented as scatter plots. *p* < 0.05 was linearly regressed to show relevant trends.

**Figure S5. Correlations between CXCR5^+^FoxP3^+^TFR cells, CXCR5^+^FoxP3^-^TFH cells, CXCR3^-^CCR6^-^TFH2 subsets and CRP among all UC patients**

Levels of CRP in active and stable remission UC patients were measured, and correlation analysis with percentages of CD4^+^CXCR5^+^FoxP3^+^ TFR cells(n=88), CD4^+^CXCR5^+^FoxP3^-^ TFH cells(n=88), CXCR3^-^CCR6^-^ TFH2 subsets(n=64) were performed among UC patients.

**Figure S6. Correlation analysis between serum cytokine concentrations and percentages of TFR and TFH cells in active UC patients**

The correlation analysis between serum IL-10, IL-12, IL-21 concentrations and percentages of CXCR5+FoxP3+ TFR cells, CXCR5+FoxP3- TFH cells were performed in 44 cases of active UC patients. Spearman’s correlation coefficients are shown. Data are presented as scatterplots. *p* < 0.05 was linearly regressed to show relevant trends.
