# Supplementary material for: The Imbalance of Circulating Follicular Helper T Cells and Follicular Regulatory T Cells Is Associated With Disease Activity in Patients With Ulcerative Colitis
Source: Front Immunol. 2020 Feb 14;11:104. doi: 10.3389/fimmu.2020.00104 (PMC7034313; doi:10.3389/fimmu.2020.00104)
Supplement: Supplementary file 2 [file Table_1.docx]

**Supplementary Table 1. Characteristics of the UC patients and healthy controls in this research**

| Characteristics | Healthy controls  (n=44) | Patients with active UC(n=44) | UC Patients in stable remission  (n=44) | *p* |
| --- | --- | --- | --- | --- |
| Male sex  (n, %) | 22(50.0) | 21(47.7) | 23(52.3) | 0.2314 |
| Age,years (median,IQR) | 45(26-56) | 46(24-57) | 41(28-58) | 0.0895 |
| WBC count  10^9^/liter(median,IQR) | 6.13(5.00-7.06) | 6.47(4.80-8.67) | 6.46(5.07-8.10) | 0.1938 |
| Lymphocyte count  10^9^/liter(median,IQR) | 1.95(1.66-2.39) | 1.94(1.49-2.29) | 1.93(1.57-2.22) | 0.0623 |
| Hemoglobin,g/liter (median,IQR) | 142(133-159) | 118(102-137) | 139(126-156) | <0.0001 |
| Serum albumin,g/liter (median,IQR) | 45.2(42.7-47.6) | 37.4(32.4-41.7) | 42.1(38.3-44.5) | <0.0001 |
| CRP,mg/liter (median,IQR) | — | 22.09(14.24-34.48) | 2.24(0.83-5.01) | <0.0001 |
| Mayo score  (median,IQR) | — | 7(5-9) | 2(1-2) | <0.0001 |

IQR,interquartile range, UC, ulcerative colitis; WBC, white blood cells; CRP, C-reactive protein
