# Supplementary figures and images for: The Imbalance of Circulating Follicular Helper T Cells and Follicular Regulatory T Cells Is Associated With Disease Activity in Patients With Ulcerative Colitis
Source: Front Immunol. 2020 Feb 14;11:104. doi: 10.3389/fimmu.2020.00104 (PMC7034313; doi:10.3389/fimmu.2020.00104)

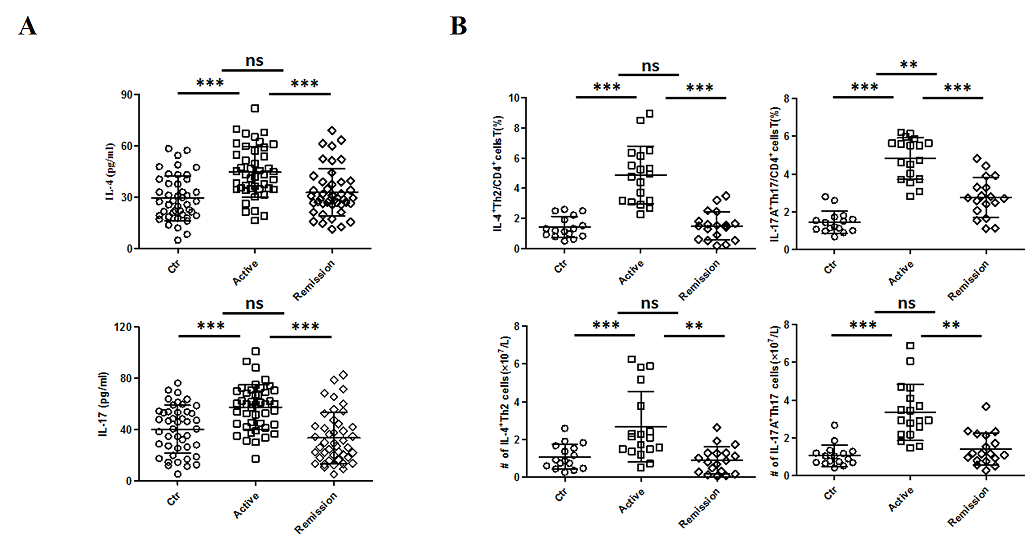

Supplement: Supplementary file 3 [file Image_1.TIF]

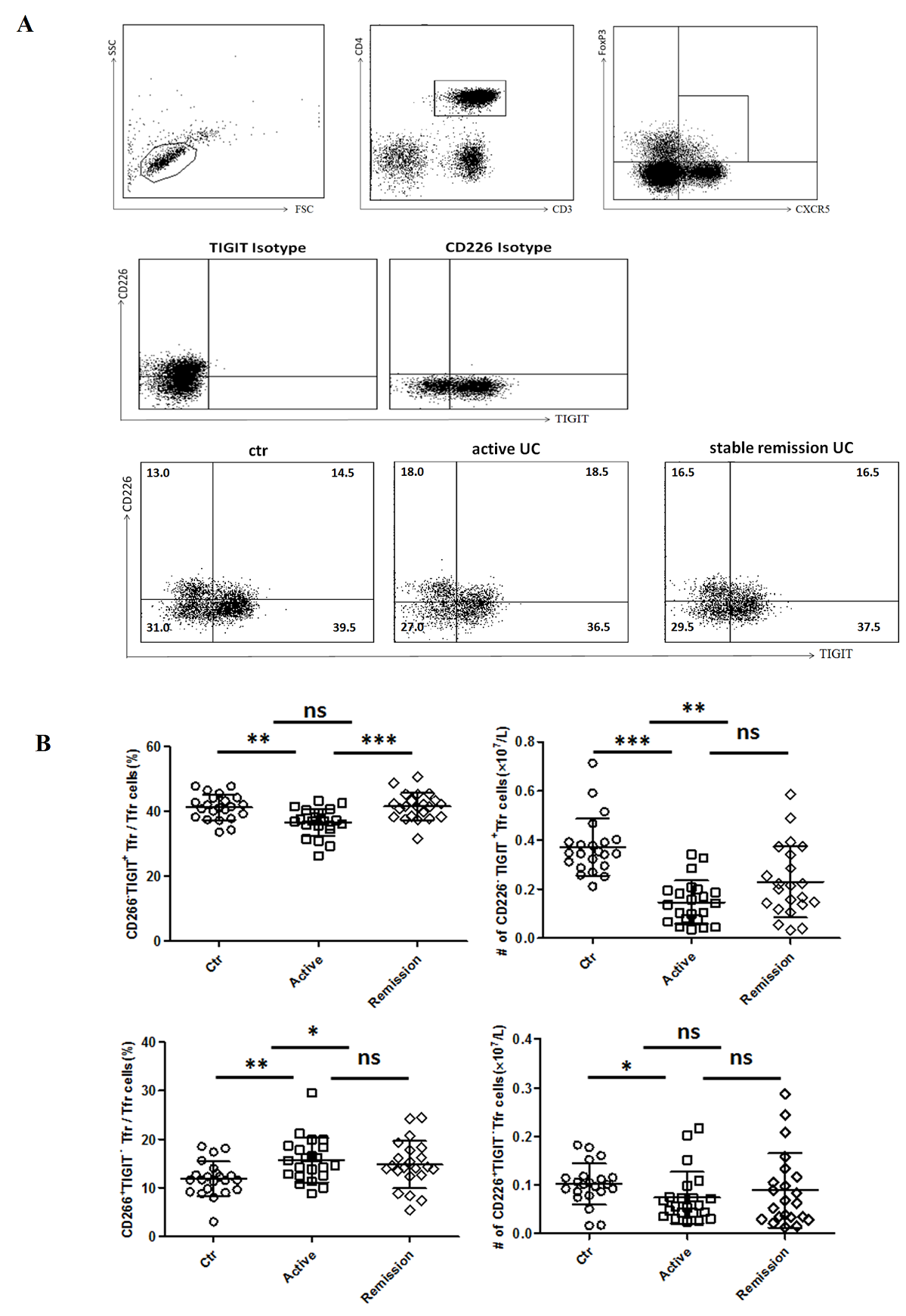

Supplement: Supplementary file 4 [file Image_2.TIF]

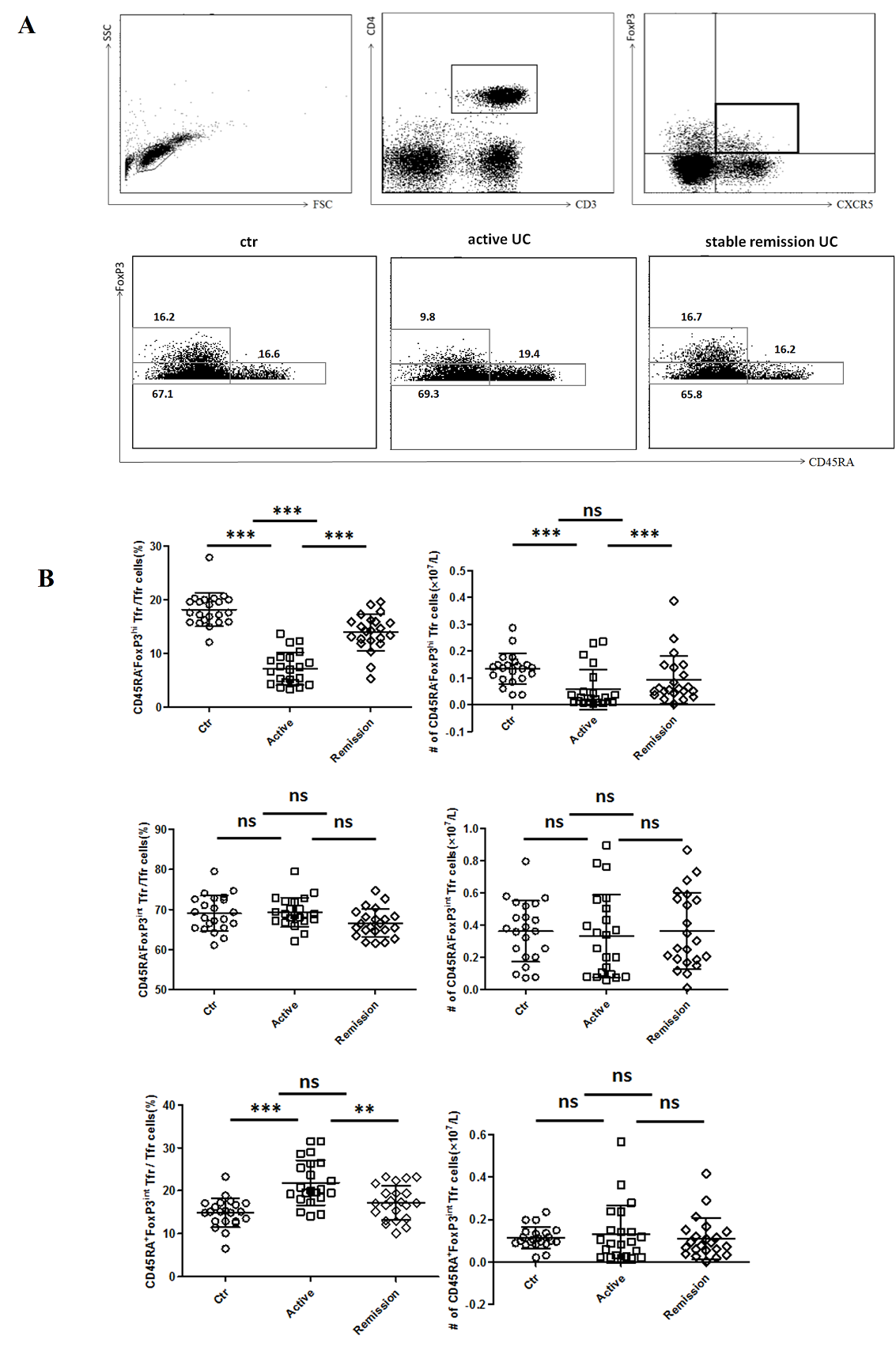

Supplement: Supplementary file 5 [file Image_3.TIF]

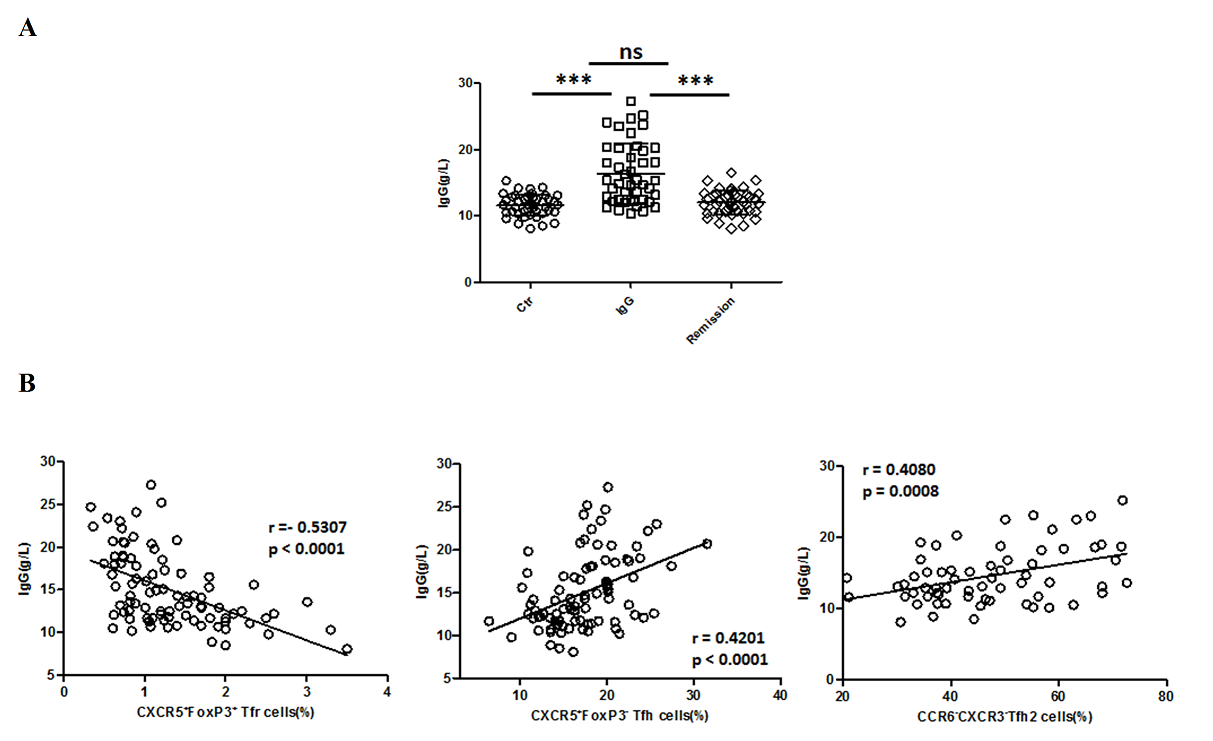

Supplement: Supplementary file 6 [file Image_4.TIF]

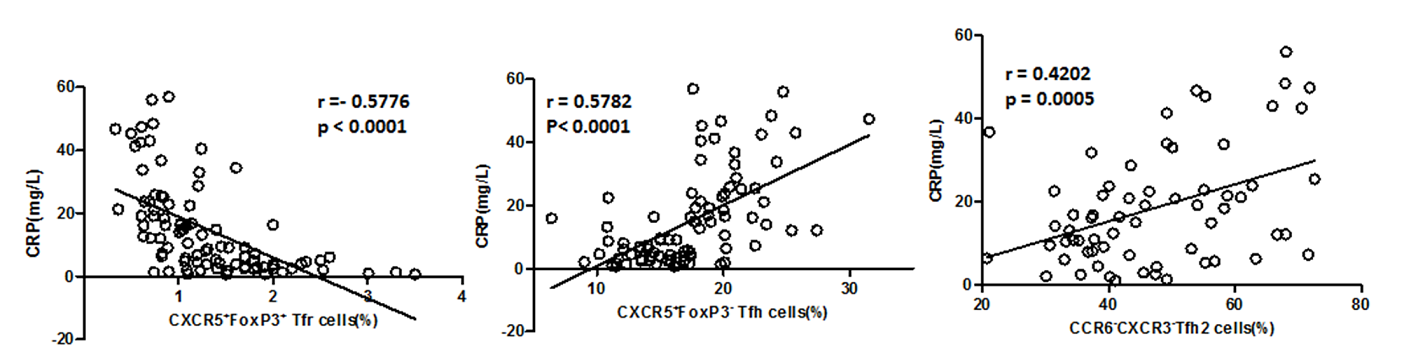

Supplement: Supplementary file 7 [file Image_5.TIF]

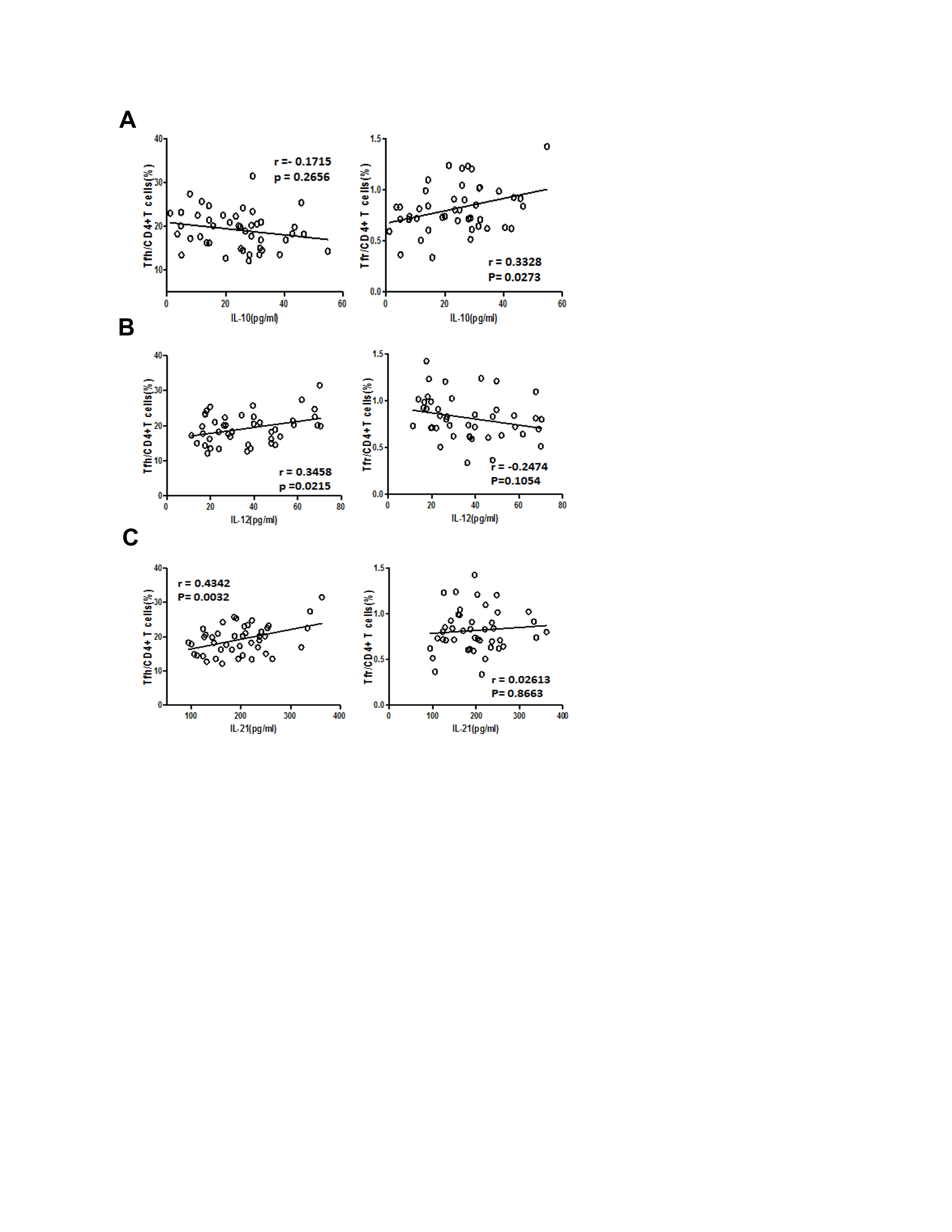

Supplement: Supplementary file 8 [file Image_6.TIF]
